# Determination of Diosmin in Pharmaceutical Products with Chemically Modified Voltammetric Sensors

**DOI:** 10.3390/ijms22147315

**Published:** 2021-07-07

**Authors:** Ramona Oana Gunache (Roșca), Constantin Apetrei

**Affiliations:** Department of Chemistry, Physics and Environment, Faculty of Sciences and Environment, “Dunarea de Jos” University of Galati, 47 Domneasca Street, 800008 Galati, Romania; oana.gunache@ugal.ro

**Keywords:** diosmin, sensor, cyclic voltammetry, pharmaceutical product

## Abstract

In this paper, the electrochemical behavior of two types of sensors based on modified screen-printed electrodes (one screen-printed electrode based on carbon (SPCE) and another screen-printed electrode modified with Prussian Blue (PB/SPCE)) was studied with the aim of sensitive detection of diosmin, an active pharmaceutical compound from the class of flavonoids. The scan electron microscopy technique was used for the morphological characterization of PB/SPCE. The preliminary analysis assessed the electrochemical behavior of SPCE and PB/SPCE in KCl solution and in a double solution of potassium ferrocyanide–potassium chloride. It was shown that the active area of PB/SPCE is superior to the one of SPCE, the greater sensitivity being related with the presence of the electroactive modifier. Similarly, in the case of diosmin detection, the PB/SPCE sensor detect more sensitivity the diosmin due to the electrocatalytic effect of PB. From the study of the influence of reaction rate on the sensor’s electrochemical response, it was shown that the detection process is controlled by the adsorption process, the degree of surface coverage with electroactive molecules being higher in the case of PB/SPCE. From the PB/SPCE calibration curve, it wasdetermined that it has high sensitivity and low detection and quantification limit values (limit of detection 5.22 × 10^−8^ M). The applicability of the PB/SPCE sensor was confirmed by sensitive analysis of diosmin in pharmaceutical products. The voltammetric method is suitable for the detection and quantification of diosmin in pharmaceutical products. The method is simple, accurate, and quick and can be used in routine analysis in the examination of the quality of pharmaceutical products and other types of samples.

## 1. Introduction

The diosmin, or 3′,5,7-trihydroxy-4′-methoxyflavone-7-rutinoside, is a flavonoid, commonly present in several plants and fruits, belonging to the *Citrus* spp. Genus, the *Rutaceae* family [1].

The flavonoid diosmin is easily obtained by dehydrogenation of the hesperidin glycoside. It was isolated in 1925 from *Scriphularia nodosa* L. and introduced into therapy in 1969 [2]. Diosmin is structurally different from hesperidin by the presence of a double bond between two carbon atoms in the C-ring of the molecule. Both contain the same carbohydrate molecule linked to the flavonoid structure. Diosmin can be produced by extracting hesperidin and hydrogenation of it [3,4,5]. In the small intestine, the diosmin is transformed, under the influence of the intestinal flora, into diosmethine, as a diosmin aglycone. This metabolite has a plasma half-life of 26 to 43 h and it is eliminated by renal excretion [6,7].

Due to diosmin’s optimal pharmacokinetic parameters, certain studies have demonstrated its antioxidative activity and reduction of high levels of free radicals produced by lead poisoning [2]. In Europe, diosmin was used on a large scale for a long time as a flebotonic and vascular protector, with oral administration in a variety of pharmaceutical products and food supplements. With a good safety profile and an important biological activity, diosmin is considered to be an optimal therapeutic option for chronic venous insufficiency (CVI), hemorrhoids, lymphedema or varicose veins [1,8]. Diosmin also has an anti-inflammatory, hypoglycemic [9], neuroprotective [10] and cardioprotective [11] effect. Several studies have demonstrated that this natural compound can induce apoptosis in various tumors [12,13,14], and the significant reduction of glioblastoma cells’ viability was also noticed, but not of healthy human astrocytes, being a natural, less expensive compound with far lower side effects compared to classical chemotherapy [15].

Given the many biological actions and the particular interest in this compound, over time, different methods have been used for the determination of diosmin, individually or simultaneously with other bioflavonoids, in various real samples, such as pharmaceuticals, biological liquids (human plasma) or plant extracts. Among the most commonly used methods are high performance liquid chromatography (HPLC) linked with an UV detector [16,17], HPLC using ionic liquids as mobile phase modifiers [18], HPLC coupled with mass spectrometry (HPLC-MS) [19], high performance thin-layer chromatography (HPTLC) [20] and reverse phase high performance thin-layer chromatography (RP-HPTLC) [21]. These methods are frequently used, and they have a very good accuracy, but they are difficult to apply because they involve extensive procedures for processing purification/extraction, followed by the analysis itself [21]. Some of the HPLC methods involve the use of strong acids and bases or other solvents and/or tertiary or quaternary mixtures in the mobile phase [22], or they involve the use of internal standards [23], all these characteristics being, in fact, disadvantages of these conventional methods [24].

It was proven to be suitable and useful for the determination of diosmin and other techniques, such as ultraviolet spectrophotometry [25,26], colorimetry [27], fluorimetry [28], infrared spectrometry [29] and voltammetry [30].

According to the literature, electrochemical voltammetric methods have been less approached compared to chromatographic or colorimetric methods; therefore, the development of electrochemical sensors may represent great interest for the rapid and specific detection of diosmin.

Table 1 shows the main types of electrochemical sensors, together with the relevant voltammetric methods and their analytical performances, reported on the studies developed for detection of diosmin.

ASV–Adsorption stripping voltammetry; SWV–Square Wave Voltammetry; DPV–Differential pulse voltammetry; AdSDPV—Adsorptive Stripping Differential Pulse Voltammetry; ZrO_2_-NPs—zirconia nanoparticles; GONRs—graphene oxide nanoribbons.

Based on this information, the purpose of this study was to investigate the electrochemical behavior and the qualitative and quantitative determination of diosmin with new voltammetric sensors based on a screen-printed carbon electrode (SPCE) and a screen-printed carbon electrode modified with Prussian Blue (PB/SPCE). After characterization of sensors and studies regarding the diosmin detection, an electroanalytical method was developed. The voltammetric method based on a PB/SPCE sensor was validated for the quantitative determination of the diosmin, using the FTIR method.

## 2. Results and Discussions

### 2.1. The Voltammetric Behaviour of the Electrodes in KCl Solution

In the first phase, the electrochemical behavior of the two screen-printed sensors, SPCE and PB/SPCE, was investigated in a solution without redox activity, KCl 10^−1^ M. The potential range was between −0.4 V and +0.7 V. The cyclic voltammograms, which were obtained with SPCE, did not have any peaks in the studied range, which reflects the lack of contamination of the electrodes surfaces and a high purity of the materials they were made of. For the PB/SPCE electrode, an anodic peak was observed at 0.196 V and a cathodic peak at 0.087 V, corresponding to the redox process of PB immobilized on an active surface of the electrode [36]. The synthesis of PB and the redox process of the PB immobilized on solid matrix is presented in Equations (1) and (2):

3[Fe^II^(CN)_6_]^4−^ + 4Fe^3+^ ⇄ Fe_4_^III^[Fe^II^(CN)_6_]_3_ (1)

Fe_4_^III^[Fe^II^(CN)_6_]_3_ + 4e^−^ + 4K^+^ ⇄ K_4_Fe_4_^II^[Fe^II^(CN)_6_]_3_ (2)

Prussian Blue Prussian White

### 2.2. The Voltammetric Behaviour of the Electrodes in Potassium Ferrocyanide-KCl Solution

In the next step, the electrodes were immersed in a solution with electrochemical activity, obtained from potassium ferrocyanide 10^−3^ M and KCl 10^−1^ M, in order to optimize the electrochemical parameters. The cyclic voltammograms were recorded at a scan rate of 0.1 V·s^−1^, in the potential range between −0.4 V and +0.7 V. This potential range was proven to be optimal in the case of potassium ferrocyanide. 

**Figure 1:**
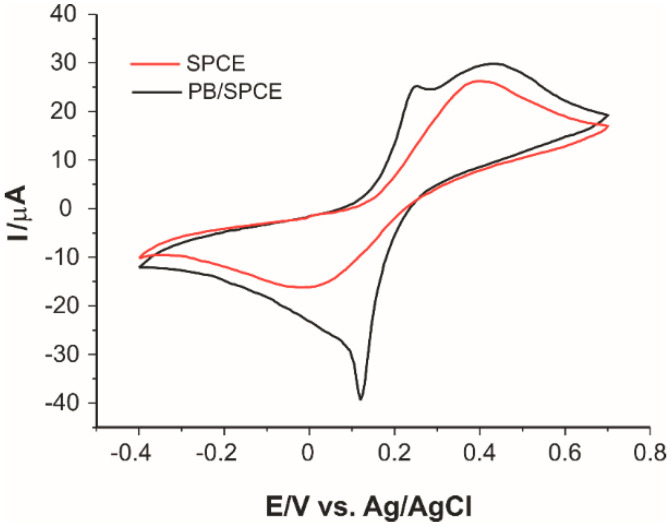
Cyclic voltammograms of the SPCE sensor (red line) and of the PB/SPCE sensor (black line) immersed in the solution obtained of potassium ferrocyanide 10^−3^ M—potassium chloride 10^−1^ M. The scan rate was 0.1 V·s^−1^.

For both sensors, anodic and cathodic peaks related to the redox processes of ferrocyanide ion at the working electrode surface could be observed. Figure 1 (red line) shows the cyclic voltammogram of the SPCE sensor in 10^−3^ M potassium ferrocyanide and 10^−1^ M KCl, recorded at the scan rate of 0.1 V·s^−1^.

In case of the immersion of PB/SPCE sensor in the double solution, two well-defined anodic peaks and a cathodic peak (Figure 1, black line) were observed. The potential range and the scan rate were at the same values as the previous analysis. The first anodic peak and the cathodic peak were corresponding to PB, while the second anodic peak was corresponding to ferrocyanide in the solution to be analyzed.

In Table 2, presented are the values of the electrochemical parameters obtained as a result of cyclic voltammograms recording for the two sensors, immersed in a potassium ferrocyanide solution 10^−3^ M and KCl 10^−1^ M.

**Figure 2:**
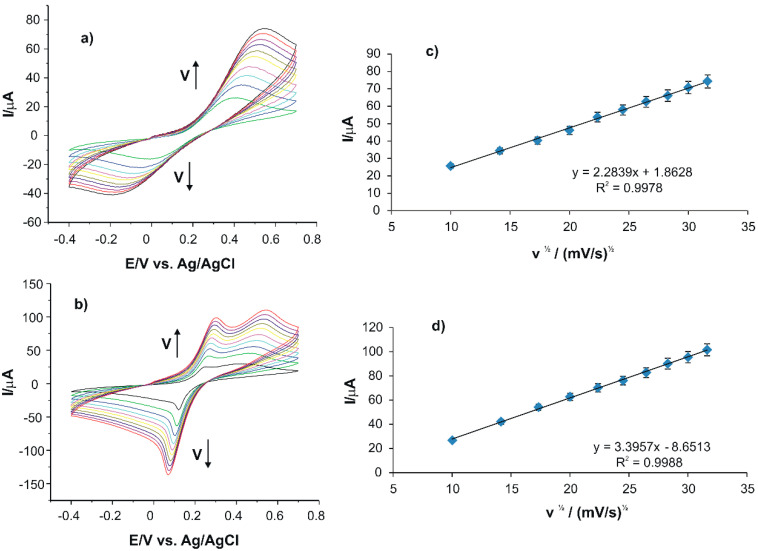
Cyclic voltammograms of the SPCE sensor (**a**) and the PB/SPCE sensor (**b**) immersed in a double solution of potassium ferrocyanide 10^−3^ M—potassium chloride 10^−1^ M, at scan rates between 0.1–1.0 V·s^−1^. The dependence between the values of the anodic current and the square root of scan rates for SPCE (**c**) and PB/SPCE (**d**).

Assessing the values of the main parameters, it can be stated that the ferrocyanide redox process is quasi-reversible [37] and that PB/SPCE presents a higher sensitivity. In the case of PB/SPCE, the peaks were more clearly defined, and a better reversibility was obtained (E and E_1/2_ have lower values). To calculate the active area of the two sensors, cyclic voltammograms were recorded in solution of potassium ferrocyanide 10^−3^ M and potassium chloride 10^−1^ M, with various scan rates in the range of 0.1–1.0 V·s^−1^. The results, which were obtained for the two sensors, are shown in Figure 2.

For redox processes of ferrocyanide/ferricyanide, it was observed that the current increased when the scan rate increased too. In order to determine which stage was limiting the rate of the electrochemical oxidation process, the dependencies between I_pa_ and the scan rate were verified, namely the square root of the scan rate. For both sensors, good linear relationships between I_pa_ and the square root of scan rate (Figure 2c,d) were obtained, with determination coefficients close to the ideal value of 1. 

According to the Randles–Sevcik equation, the peak current is directly proportional to the analyte concentration, to the square root of the diffusion coefficient and the square root of the scan rate. In the case under consideration, the linear relation between I and v^1/2^ proves that the redox process is controlled by the diffusion process of the active species [38].

To calculate the active area of the electrodes, the Randles–Sevcik equation was used, due to the linear dependence of I_pa_ and v^1/2^. In cyclic voltammetry, the Randles–Sevcik equation describes the effect of scan rate on the redox peaks, according to Equation (3):

(3) $$I_{pa}=268600\times n^{3/2}\times A\times D^{1/2}\times c\times v^{1/2}$$

where:

*I_pa_*—anodic peak current (A);
*n*—number of electrons transferred in the redox process, 1 for ferrocyanide ion;
*A*—electrode area (cm^2^);
*D*—coefficient of diffusion (cm^2^·s^−1^);
*c*—concentration (mol·cm^−3^);
*v*—scan rate (V·s^−1^);

According to the literature, the diffusion coefficient of the ferrocyanide ion is D = 7.26 × 10^−6^ cm^2^·s^−1^ [39].

The linear regression equations, the coefficients of determination (R^2^), the areas of active surface and the roughness coefficients are centralized in Table 3. The geometric area of the sensors was 0.125 cm^2^.

Both electrodes had a higher value of the area of the active surface than the geometric area, which indicates that both presented a good sensitivity and could be successfully used to detect bioflavonoids such as diosmin, the compound of interest in this study. However, the superiority of PB/SPCE was proven, having a roughness coefficient of 3.75 and an area of the active surface approximately 4 times bigger than the geometric area. These results were related to the higher peak current values.

This high sensibility of the PB/SPCE was due to the changes in the sensors’ surface, which promoted the transfer of electrons and the accumulation or diffusion of analytes. The literature provides numerous studies in which the modified sensors with Prussian Blue present better characteristic and better results in the detection of antioxidant compounds [40].

### 2.3. The Study of Voltammetric Detection of Diosmin

For quantitative determination of diosmin in two pharmaceutical products (Detralex and Fluxiv), SPCE and PB/SPCE were used. The results were compared with those obtained by the conventional method, FTIR [29].

**Figure 3:**
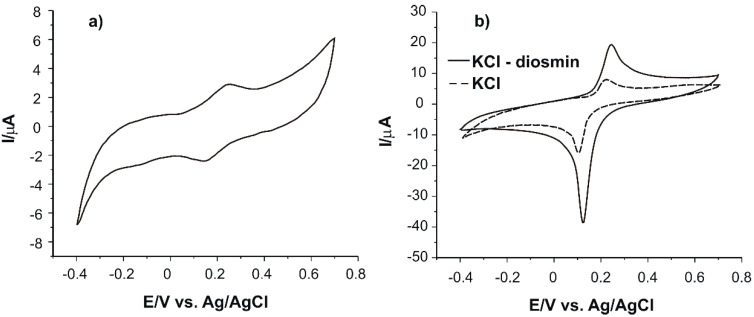
(**a**) CV of SPCE immersed in a double solution of 10^−3^ M diosmin/KCl 10^−1^ M; (**b**), CVs of PB/CSPE immersed in 10^−4^ M diosmin/KCl 10^−1^ M solution (solid line) and in KCl 10^−1^ M solution (dashed line).

For the electrochemical analysis, an extraction of diosmin was performed, using the solid–liquid method, from the pharmaceutical product Detralex, and a solution of 10^−4^ M diosmin (in KCl 10^−1^ M) was prepared. The obtained solution was added in the electrochemical cell for electroanalysis. The potential range used was between −0.4 and +0.7 V, and the scan rate was 0.1 V·s^−1^. The two working electrodes were used one at a time, and the currents and potentials of the observed peaks (Figure 3), together with the values of some calculated electrochemical parameters, are given in Table 4.

**Figure 4:**
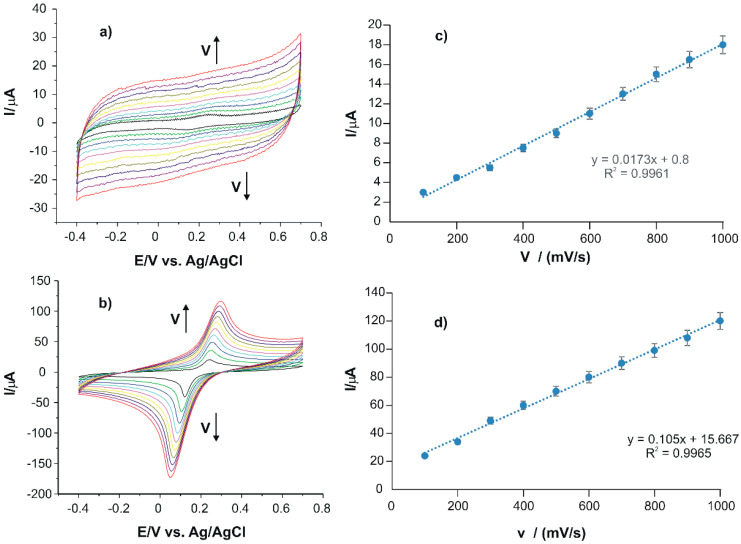
Cyclic voltammograms of SPCE (**a**) or PB/SPCE (**b**) immersed in a double solution 10^−4^ M diosmin/KCl 10^−1^ M, recorded with various scan rates in the range 0.1–1.0 V·s^−1^. The dependence between the values of the current of the anodic peak and the scan rate for SPCE (**c**) and PB/SPCE (**d**).

In the case of SPCE, an anodic and a cathodic peak were observed, due to the redox process of diosmin [33]. In the case of PB/SPCE, the peaks which were observed corresponded to the overlap of the redox processes of diosmin and PB (Figure 4b) [40]. It could be noticed that in the case of PB/SPCE, the peaks had higher currents and lower E_1/2_. As shown in Figure 4, in the case of PB/SPCE, the potentials of the peaks had lower values, which indicates that the redox process was electrocatalyzed by the PB, which makes the PB/SPCE sensor more sensitive than SPCE [41].

**Scheme 1:**
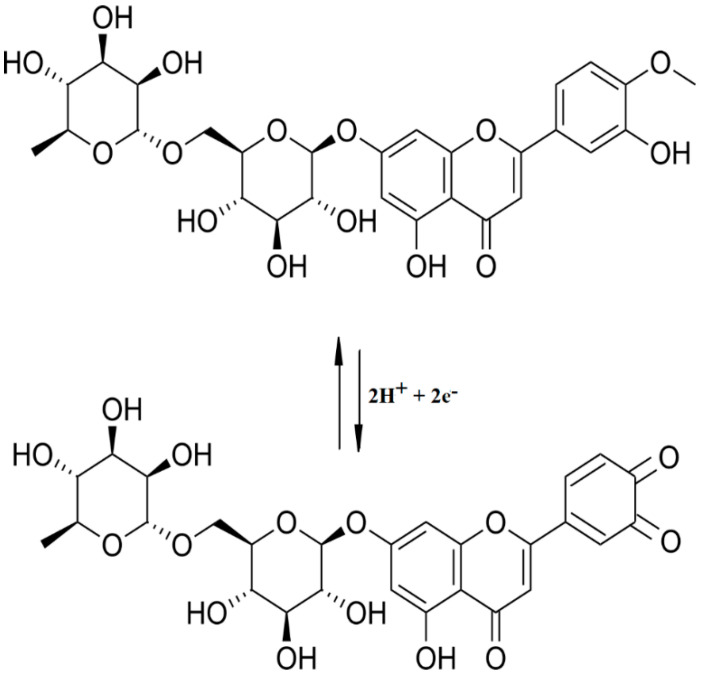
The mechanism of the voltammetric detection of diosmin.

The mechanism of diosmin detection by the two sensors is shown in Scheme 1 [33].

Thus, in the case of SPCE, at the anodic scan, it occured at the oxidation of diosmin to the ortho-quinonic derivative, followed by the reduction at the cathodic scan [33]. With regards to PB/SPCE, the redox process of diosmin was catalyzed by PB, and for this reason, the anodic and cathodic peaks were more intense than in the case of SPCE [42].

For the next step, the kinetics of redox process was performed by recording the cyclic voltammograms with various scan rates between 0.1 and 1.0 V·s^−1^. For both sensors, the intensities of the anodic and cathodic peaks increased simultaneously with scan rate increasing (Figure 4a,b).

Figure 4b,c shows the dependence between the intensity of the anodic peaks and scan rates, obtaining a linear dependence between I_pa_ and v. This demonstrates that the redox process was controlled by the adsorption process.

Considering the linear dependence among the currents of the anodic peak and the scan rate, the degree of coverage of the electrode surface (Γ) from the Laviron’s equation (Equation (3)) [43] was calculated for both sensors:

(4) $$i_{pa}=\frac{n^{2}F^{2}\Gamma Av}{4RT}$$

where:

Γ—surface coverage, mol·cm^−2^;
*i_pa_*—the current of the peak, A;
*A*—electrode surface, cm^2^;
*n*—the number of electrons transferred during the redox processes, (two for diosmin);
*F*—Faraday’s constant, 96,485 C·mol^−1^;
*R*—universal gas constant, 8.314 J·mol^−1^·K^−1^;
*T*—absolute temperature, K.

The values of Γ which were obtained were in agreement with those reported in literature for voltammetric sensors, being 7.14 × 10^−10^ mol·cm^−2^ for SPCE and 2.1 × 10^−9^ mol·cm^−2^ for PB/SPCE [44,45]. It can be noticed that the obtained value for PB/SPCE was much higher, with an order of magnitude comparative with the value obtained for SPCE, which explains the higher sensitivity of PB/SPCE to the voltammetric detection of diosmin [46].

### 2.4. The Voltammetric Detection of Diosmin in the Pharmaceutical Product Fluxiv

**Figure 5:**
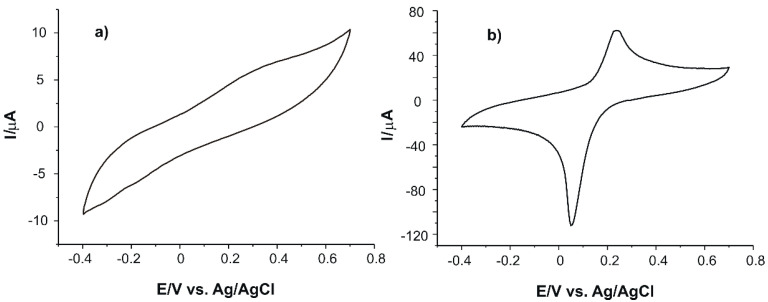
Cyclic voltammograms of sensors SPCE (**a**) and PB/SPCE (**b**) immersed in a solution obtained from the pharmaceutical product Fluxiv, recorded at the scan rate of 0.1 V·s^−1^.

In order to study the detection of dopamine in the case of the pharmaceutical product Fluxiv, the tablets were triturated; then, a quantity was dissolved in KCl 10^−1^ M. The mixture was homogenized by means of an ultrasound bath and filtered. The obtained solution was analyzed with both sensors, SPCE and PB/SPCE. The range of potential and the scan rate were the same as in the case of diosmin analysis. The obtained cyclic voltammograms are shown in Figure 5.

In the case of SPCE, a large anodic peak (E_pa_ = 0.285 V, I_pa_ = 5.66 µA) was observed. The PB/SPCE sensor presented an anodic (E_pa_ = 0.235 V; I_pa_ = 61.9 µA) and a cathodic (E_pc_ = 0.050 V; I_pc_ = −111.78 µA) peak. In both cases, the peaks were related to the presence of diosmin, but with the other active compounds from the pharmaceutical product. From this data, it can be concluded that the PB/SPCE sensor has a higher sensitivity than SPCE, with the current of the anodic peak being 10 times higher.

### 2.5. The Study of Voltammetric Detection of Diosmin in the Pharmaceutical Product Fluxiv

The calibration curve for the PB/SPCE sensor was carried out by recording of cyclic voltammograms in diosmin solutions with various concentrations, KCl 10^−1^ M, adding measured volumes of the stock solution 10^−3^ M diosmin in an exactly measured volume of 10^−1^ M KCl solution (blank solution). 

**Figure 6:**
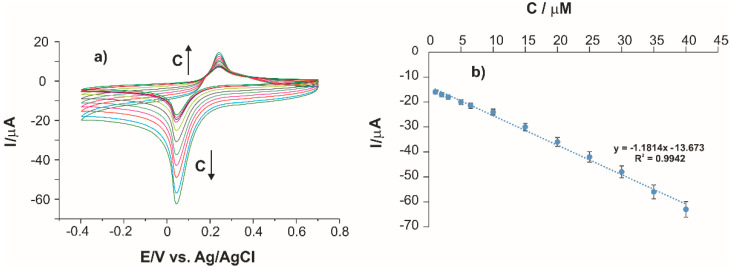
(**a**) CV of PB/SPCE immersed in diosmin solutions with various concentrations (in the range of 1–40 µM); (**b**) calibration curve based on the cathodic peak.

The responses of the PB/SPCE sensor in diosmin solutions of various concentrations are shown in Figure 6. It can be observed that upon the increase of the diosmin concentration, the intensities of anodic and cathodic peak also increased.

For the development of the calibration curve, the linear dependence was represented between the currents of cathodic peak and the diosmin concentrations in the analyzed solutions.

A linear dependence was observed between the intensity of the cathodic peak and the diosmin concentration in the range of concentrations between 1 and 40 µM (Figure 6b). The equation of calibration and R^2^ are presented in Figure 6b. Using the equation of the calibration, the values of the limit of detection (LOD) and limit of quantification (LOQ) were calculated in agreement with Equations (5) and (6) [47]:

LOD = 3σ/m (5)

LOQ = 10σ/m(6)

where σ is the standard deviation of the electrochemical signal for the blank sample to the corresponding peak potential of the diosmin reduction, and m is the slope of the calibration curve.

The limit of detection and the limit of quantification for the PB/SPCE sensor at the detection of diosmin had the values of 5.22 × 10^−8^ M (LOD) and 1.74 × 10^−7^ M (LOQ). The values obtained with the PB/SPCE sensor were comparable or lower, compared with other sensors reported in the literature (Table 1). 

The sensor PB/SPCE presented small values of LOD and LOQ, which indicates the higher sensitivity of the sensor developed in this study and demonstrates the feasibility of the voltammetric method for the analysis of diosmin in pharmaceutical products. The obtained values were better or similar to those reported in the literature, which demonstrates that the PB/SPCE sensor had adequate electroanalytical performances to detect the diosmin in real samples.

### 2.6. Sensor Repeatability and Reproducibility

The repeatability of the sensor response was studied. The cyclic voltammograms were registered in a 10^−4^ M diosmin solution. Among replicate cyclic voltammograms, the sensor was rinsed with ultrapure water and dried in a desiccant. The relative standard deviation (RSD) of seven replicate measurements, taking into account the cathodic peak current, was 2.7%. 

The reproducibility of the sensor fabrication was studied by preparing five sensors in the same conditions (stock solution, added volume, drying time, storage conditions) and registering the cyclic voltammograms in a 10^−4^ M diosmin solution. The RSD of cathodic peak currents corresponding to diosmin was 3.5%, demonstrating the control of the sensor preparation.

### 2.7. The Quantification of Diosmin in Pharmaceutical Products

For the determination of diosmin in pharmaceutical products, the PB/SPCE sensor was used, and the cyclic voltammograms were recorded in solutions which contained the mentioned pharmaceutical products. Based on the current of the anodic peak, the number of tablets dissolved in a KCl 10^−1^ M solution and the equation of the calibration, the quantity of diosmin of pharmaceutical products was calculated, and the results were included in Table 5. The obtained results were in good agreement with the values reported by the producers, which demonstrates the PB/SPCE sensor accuracy and the lack of interference.

**Figure 7:**
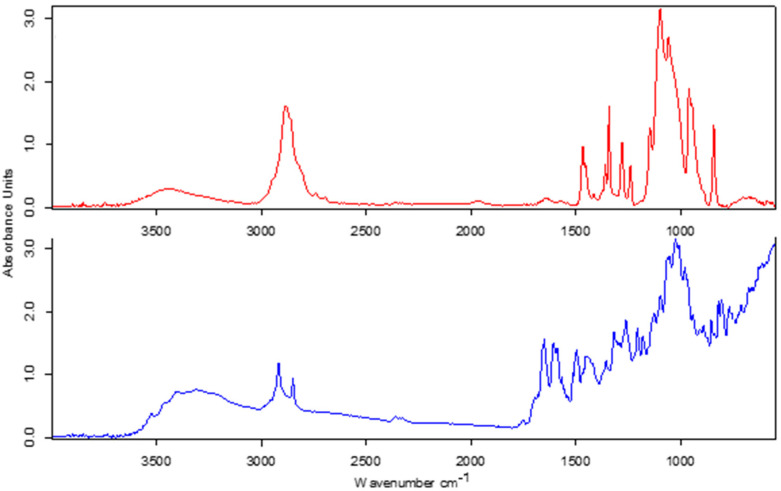
FTIR normalized spectra of Detralex (red line) and Fluxiv (blue line).

To validate the voltammetric method, the two pharmaceutical products were analyzed using the infrared spectrometry method. FTIR method presents a huge potential, and it is widely used in pharmaceutics quality control, because it can confirm the identity and quantity of the active compounds from pharmaceutical products [48,49]. The FTIR spectra of the analyzed pharmaceutical products are presented in Figure 7. Compared to the pure diosmin FTIR spectrum, those of pharmaceutical products were more complex, due to the presence of other pharmacological active compounds and excipients.

For diosmin quantification, five solid diosmin-KBr standards of different concentration were prepared, in the range of 100–500 mg/g, and the linear calibration model was performed based on the peak absorbance from 1097.64 cm^−1^. From the peak absorbances at 1097.64 cm^−1^, obtained for the pharmaceutical products, and using the equation of the calibration (A = 0.0031 × c, R^2^ = 0.9933), the quantity of diosmin was calculated. The results are presented in Table 5.

From Table 5, it can be observed that the results obtained by the two methods and product labels were in good correlation. Therefore, it can be concluded that the obtained results with the PB/SPCE at diosmin quantification were accurate, and the voltammetric sensor was validated at a laboratory level.

## 3. Materials and Methods

### 3.1. Reagents and Solutions

Potassium chloride, sodium hydroxide and potassium ferrocyanide used in the preliminary studies were acquired from Sigma-Aldrich (St. Louis, MI, USA), having the highest analytical purity. Iron(III) hexacyanoferrate(II) (Prussian Blue) used for the modification of electrodes was purchased from Sigma-Aldrich. All experiments were performed in aerobic conditions at room temperature. All solutions have been prepared using ultrapure water, MilliQ water (resistivity 18.2 MΩ·cm), obtained with a Milli-Q Simplicity^®^ system.

Diosmin used as standard in this study was extracted from the pharmaceutical product Detralex. The extraction of diosmin was carried out from the powder obtained from the pharmaceutical product Detralex, which contains 90% diosmin (450 mg) and 10% hesperidin (50 mg) as pharmacologic active principles, with ultrapure water by solid–liquid extraction using a chromatographic column made from glass. The extraction was based on the difference of the solubility in water of these compounds. The total extraction of the diosmin was checked by registering the UV spectrum of the effluent solution. The liquid extract was dried in a rotary evaporator system, and the solid powder obtained was characterized by UV spectroscopy, melting point determination and FTIR spectrometry. The max obtained was 268 nm; the melting point was 276–277 °C. These results are in agreement with those reported in literature for pure diosmin [26,50]. Furthermore, the vibrational peaks characteristics for diosmin were identified in FTIR spectrum [29].

The preparation of the 10^−3^ M diosmin stock solution included the weighting of an appropriate amount of solid diosmin, dissolving it in 2 mL of 0.2 M NaOH and completing to 10 mL with ultrapure water. For the measurements, the appropriate volumes of stock solution were mixed with 0.1 M KCl solution, which was a support electrolyte in the voltammetric measurements. The stock solution was prepared daily and kept at 4 °C before analysis.

### 3.2. Electrochemical Measurements

**Figure 8:**
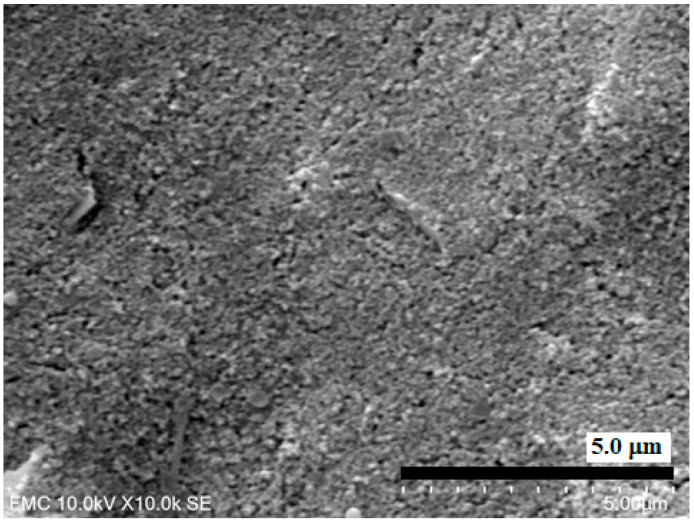
SEM image of the sensitive element of SPCE modified with PB.

The electrochemical measurements were conducted with an EG&G potentiostat–galvanostat (Princeton applied Research, Oak Ridge, TN, USA), connected to the Echem specialized software for data acquisition, experimental parameter setting and interpretation of cyclic voltammograms. The solutions to be analyzed were placed in an electrochemical cell of 50 mL, with three electrodes. The reference electrode was the silver/silver chloride (KCl 3M) electrode, and the counter electrode was a platinum wire. Screen-printed electrodes (SPCE) were purchased from Metrohm DropSens (Oviedo, Spain). A screen-printed electrode modified with Prussian Blue (PB/SPCE) was developed by the casting method using 10 µL of PB (10 mg PB/1 mL water). The 10 µL were easily and precisely deposited with a Hamilton syringe and using a magnifier for a better view of the working electrode. The quantity of PB solution was optimized in the sense that the surface was completely covered with the solution. The deposition of the PB solution was carried out in two steps; in each 5 µL were casted on the working electrode surface. After each addition, the evaporation of the water was performed for 2 h in a desiccator at room temperature. The modified sensors were kept at 4 °C in a closed box before electroanalysis. The morphology of the sensitive element of PB/SPCE was carried out by scanning electron microscopy (SEM) (Scanning Electron Microscope FlexSEM 1000, Hitachi, Tokyo, Japan). The SEM image of the sensor surface is presented in Figure 8.

The PB/SPCEs are disposable, but they could be used for more than 50 measurements in the electrolyte solutions without significant loss of their responses. After each measurement, the sensors were removed from the solution, rinsed with ultrapure water and kept in a laboratory fridge at 4 °C in a closed box in the absence of the light.

The values’ peak potentials observed in cyclic voltammograms were reported in relation to the potential of the reference electrode Ag/AgCl (KCl 3M). The cyclic voltammograms were recorded in an optimal potential range for these electrodes, in the range between −0.4 V and +0.7 V. For the stabilization of the sensors’ signals, six successive cycles were recorded by cyclic voltammetry with a scan rate of 0.1 V·s^−1^. 

The FTIR spectra were obtained with a Bruker ALPHA FT-IR (BrukerOptik GmbH, Ettlingen, Germany) spectrometer, which uses OPUS (BrukerOptik GmbH, Ettlingen Germany) software in the range of 4000–500 cm^−1^ (32 scans, 4 cm^−1^ resolution) in the total attenuated reflected mode (ATR). Between the measurements, the ZnSe crystal was cleaned with ultrapure water and isopropanol. The spectra were recorded in relation to the air (background). 

### 3.3. The Analysis of Pharmaceutical Samples

The pharmaceutical products chosen for the analysis were purchased from local pharmacies, based on a medical prescription, having them as tablets with the active compound, diosmin. Fluxiv is a combination of three flavonoids containing 180 mg diosmin, 20 mg hesperidin, 300 mg troxerutin and 100 mg vitamin C. All these components act synergistically, reinforcing each other’s antioxidant and vasoprotective actions. It is an OTC (over-the-counter) product, which can be dispensed without a medical prescription. 

Another product, Detralex, contains 500 mg of purified flavonoid fraction, equivalent to 450 mg of diosmin and 50 mg hesperidin. Detralex is a product which can be released only with a medical prescription, which will be retained in the pharmacy (P-6L). It is recommended in vascular conditions such as varicose vein or hemorrhoidal disease.

The weighting of the compounds and pharmaceutical products was carried out using an analytical balance AS60/220. R2 (SC Partner Corporation SRL, Bucharest, Romania). In order to analyze the diosmin in the pharmaceutical products, the tablets were triturated in a mortar until a fine, homogeneous powder was obtained. A weighted amount of powder was dissolved in 10^−1^ M KCl aqueous solution. The solution was prepared using the Elmasonic S10H (Elma Schmidbauer GmbH, Singen, Germany) device for complete dissolution of the compounds and solution homogenization. The resulting solutions were filtered, diluted and then inserted into the electrochemical cell, and the three electrodes were connected (working electrode, reference electrode and counter electrode). The estimated concentrations of diosmin in the solutions to be analyzed were around 20 µM. All pharmaceutical samples were analyzed in triplicate. All experimental procedures and protocols were in line with the European Communities and Council Directive of 24 November 1986 (86/609/EEC).

## 4. Conclusions

This paper demonstrated the validity of a sensor based on a screen-printed carbon electrode modified with Prussian Blue to detect and quantify diosmin. The redox process of diosmin at the surface of the modified sensor was electrocatalyzed by PB, obtaining a sensitivity in the nanomolar range. To validate the voltammetric method, pharmaceutical products were analyzed with the sensor and with the infrared spectrometric method, and the results obtained by both methods were in agreement and correlated with diosmin quantities reported by producers. The sensor developed in this study is adequate for the detection of diosmin in pharmaceutical products, and the method can be extended to other products containing diosmin. The method is easy to implement, has adequate sensitivity, requires a small volume of the sample and has a low cost.

## Figures and Tables

**Table 1:** The main voltammetric sensors used in diosmin detection.

Sensor	Detection Technique	Linear Range	LOD	Reference
Glassy carbon electrode	ASV	5.0 × 10^−8^–9.0 × 10^−6^ M	3.5 × 10^−8^ M	[30]
Carbon paste electrode	SWV	12.5–200 µM	2.663 × 10^−6^ M	[31]
Carbon paste electrode	SWV	−	2663 nM	[32]
ZrO_2_-NPs-coated poly(diallyldimethylammonium chloride)-functionalized graphene modified electrode	DPV	5 × 10^−9^–2 × 10^−6^ M	2 × 10^−9^ M	[33]
GONRs prepared by unzipping of multiwalled carbon nanotubes	DPV	51.01–39.21 μM	0.015 μM	[34]
	SWV	25–3.48 μM	0.049 μM	
Pencil Graphite Electrode	DPV	1.00 × 10^−6^–1.00 × 10^−5^ M	2.76 × 10^−7^ M	[35]
	AdSDPV	1.00 × 10^−7^–2.50 × 10^−6^ M	7.42 × 10^−8^ M	

**Table 2:** Electrochemical parameters obtained from the cyclic voltammograms of the sensors immersed in K_4_[Fe(CN)_6_] 10^−3^ M- KCl 10^−1^ M solution.

Sensor	E_pa_ (V)	E_pc_ (V)	E_1/2_ (V)	ΔE (V)	I_pa_ (µA)	I_pc_ (µA)	I_pc_/I_pa_
SPCE	0.397	−0.059	0.228	0.456	25.901	−15.971	0.616
PB/SPCE	0.245	0.025	0.110	0.220	24.422	−38.93	1.592
	0.451	-	-	-	30.112	-	-

E_pa_—potential of the anodic peak; E_pc_—potential of the cathodic peak; I_pa_—current of the anodic peak; I_pc_—current of the cathodic peak; E_1/2_—half-wave potential, E = E_pa_ − E_pc_.

**Table 3:** The linear fitting equations (I_pa_ vs. v^1/2^), active areas and roughness coefficients.

Sensor	Equation	R^2^	Active Area (cm^2^)	Roughness Coefficient
SPCE	I_pa_ = 2.2839 v^1/2^ + 1.8628	0.9978	0.315	2.52
PB/SPCE	I_pa_ = 3.3957 v^1/2^ − 8.6513	0.9988	0.469	3.75

**Table 4:** Electrochemical parameters obtained by analyzing the double 10^−4^ M diosmin/KCl 10^−1^ M solution.

Electrode	E_pa_ (V)	E_pc_ (V)	E_1/2_ (V)	I_pa_ (µA)	I_pc_ (µA)	I_pc_/I_pa_
SPCE	0.245	0.141	0.193	2.897	−2.490	0.859
PB/SPCE	0.239	0.119	0.179	19.835	−40.358	2.034

**Table 5:** The results of diosmin quantification in the two pharmaceutical products.

Pharmaceutical Product	Diosmin Quantity/mg per Tablet		
	Sensor Method	FTIR Method	Product Label
Detralex	454 ± 2	458 ± 5	450
Fluxiv	183 ± 1	184 ± 2	180

## Data Availability

The authors confirm that the data supporting the findings of this study are available within the article.
